# Supplementary material for: Trend and associated factors of cesarean section rate in Ethiopia: Evidence from 2000–2019 Ethiopia demographic and health survey data
Source: PLoS One. 2023 Mar 16;18(3):e0282951. doi: 10.1371/journal.pone.0282951 (PMC10019649; doi:10.1371/journal.pone.0282951)
Supplement: Attachment — Submitted filename: Response to Reviewers.docx [file pone.0282951.s001.docx]

**Rebuttal letter**

ManuscriptID: D-22-08008

Manuscript Title: Trend and associated factors of cesarean section rate in Ethiopia: evidence from 2000-2019 health survey of Ethiopia

I thank the editor and two reviewers very much for their constructivecomments and suggestions on our manuscript. (To show) Below is our response to each point raised by the academic editor and reviewers.

The authors included a point-by-point response within the 'Response to Reviewers' box in the submission system and modifications are highlighted changes made to the original version on the main manuscript

The authors addressed all the issues raised by the Reviewers and the Editor. I hope the response satisfied you all. The revisions significantly improved the manuscript.

Sincerely

On behalf of all the author

RahelMezemir.

**Academic editor**

1. **Please ensure that your manuscript meets PLOS ONE's style requirements, including those for file naming**

Response: Thank you very much for the(your)suggestion, the file(name was) changed to adhere to the style of the requirement. Hopefully, there are no longer any deviations from the style criteria.

2. **We suggest you thoroughly copyedit your manuscript for language usage, spelling, and grammar**.

**Response**: thank you again for yoursuggestion, thelanguage usage, spelling, and grammar wereamended and highlighted with yellowcolour on the main manuscript in the file name “support information

**Reviewer reports:
Reviewer #1:**

1. **I think the authors should not mention which software ,they used for estimation in the abstract.**

**Response**:The author’s would like to thank this reviewer for his constructive comments and suggestion. The suggestion was well accepted the authors removed Extended Mantel-Haenszel and STATA 14.1 software from the abstract of the main manuscript

2. **In the last paragraph of the introduction, please mention the value addition of the study.**

Response:***:*** Again the author’s would like to thank the reviewer for his critical reviewing the relevance of our work, The authors tried to list a few pointson the additive value of this study, for instance, the study observing 24-year trend data which highlights national level characteristics.This could lead to policy decisions at the national level and it helps the concerned body that seekslarge data (page number 5 and line number 99). Another additive value of this study.included important factors which were not listed in another study that was associated with cesarean section ratelike;,the size of the baby (very large, average, and small )and parity.

3. **Please add another section of the literature review after the introduction.**

**Response**: The authors acknowledge the suggestion was given from the review, but it did not meet PLOS ONE's manuscript style requirements. According to PLOS ONE the styleofrequirement literature review is part of the introduction and authors follow this guideline ;thus, the literature review is included in the main manuscript at end of the introduction ( page 4, lines number 72-90).

4. **Why did you use this methodology, and method of estimation, please give a concrete justification, similarly, why did you select these variables, please explain in detail**.

**Response**; I would like to thank the reviewer'squestion, it is an important concern.

Briefly, the study was performed using cross-sectional data obtained from the Ethiopia demographic and health survey (EDHS) conducted in 2000, 2005, 2011,2016, and 2019. These all were nationally representative household surveys conducted by the Central Statistical Agency of Ethiopia. Therefore, the reason for using this methodology in the current study was:

I. The EDHS is the only source of nationally representative household surveys that provide a wide range of data on several health indicators including cesarean sections.
II. The current study investigatesdata starting from 1995 to 2019year, which was a 24-year trend of cesarean section rate in Ethiopia,thus, this kind of big data is difficult toaccess throughthe primary sourcedata orchart review. Furthermore, this kind of national datais performed meticulously to give sufficient statistical power for analysis.
III. As we explained previously, the data is big and conducted from eleven regions and two municipal governments of Ethiopia, so all the data is aresummarized in one big figure and making it easy for the reader to look into the whole picture of the problemand to identify the gap.

**Response**; The reason for the selection of these variables that used in the current study was

firstly, the author search different literature by using the following major database, PUBMED/MEDLINE, HINARI, Google scholar and the key terms used in PubMed search wascesarean section OR abdominal surgery ” AND “ trend OR pattern OR magnitude OR prevalence OR distribution ‘’AND” associated factors “AND “Ethiopia OR Africa OR/ world. after searching all this literature we selected the statistically significant variable. And then the author used two strategies to select these variables:

A. individually choose the independent variable based on their relationship with or influence on the dependent variable
B. and choose independent variable based on their contribution to the model’s overall performance in explaining the variance of and forecasting the dependent variable

Finally, before running the final model, multicollinearity was assessed by using the variance inflation factor. Variables highly correlated with other independent variables were excluded from the final multinomial logistic regression model.

5. **Always compare and contrast your findings with the existing literature and elaborate are they similar to the previous studies or if there is any difference and how and why?**

**Response**; The author’s thank the Reviewer for highlighting this point, the author made extensive amendmentstothe discussion part and saw in detail in the main manuscript.

6. Conclusions need serious consideration. Please rewrite it, and also add policy implications, limitations of the study, and way forward.

**Response**; the author rewrite the conclusion and added policy implementation in the main document of the manuscript, but the author put the limitation of study at end of the discussion part(page 30, line number 413-41) becausethere is no separate heading for the limitation of the study in PLOS ONE manuscript guideline.

**Reviewer #2**

1. **The paper is very poorly structured. The authors stated that they applied a multilevel logistic regression model but I did not find the model in the paper. I did not find any of which variables are used at the first level, second level, and so on. They have not mentioned how many level variables were used in this study. The authors give unnecessary details of data.**

**Response;** Thank you very much for your constructive question and suggestion, (that showsthe model belew the description for the question.

The four models were carried outfor factors related to CS rate. The first model was an empty model that was used to calculate the random variability in the intercept and there was no variable in this model. The second model estimated the influence of individual-level factors on CS delivery. Variables like; the mother's age, ANC visit educational level, wealth quantile, BMI, parity, and birth order are included in model II ;

The third model looked at how community-level factors are associated with CS delivery. The variable included in model III;are residence and region.

The fourth model computed the influence of individual and community-level factors on cesarean delivery. The variable included in model IV is;the mother’s age at birth, education, BMI, parity, birth order, ANC visit, type of pregnancy, residence, and geographical region. the detail description (page 24-25), line number 331, ,Table 4. Multilevel multivariable logistic regression output for cesarean section rate among live births in Ethiopia 2016)
