# Supplementary material for: Trend and associated factors of cesarean section rate in Ethiopia: Evidence from 2000–2019 Ethiopia demographic and health survey data
Source: PLoS One. 2023 Mar 16;18(3):e0282951. doi: 10.1371/journal.pone.0282951 (PMC10019649; doi:10.1371/journal.pone.0282951)
Supplement: Attachment — Submitted filename: Response to Reviewers.docx [file pone.0282951.s002.docx]

**Rebuttal letter**

Manuscript ID: D-22-08008

Manuscript Title: Trend and associated factors of cesarean section rate in Ethiopia: evidence from 2000-2019 health survey of Ethiopia

I thank again the editor and two reviewers very much for their response and suggestions on tour manuscript. To show our response to each point raised by the editor as below.

The authors included the response within the response to reviewers' box in the submission system and modifications are highlighted to changes the original version of the main manuscript.

The authors addressed all the issues raised by the Editor. I hope that the response satisfied you all. The revisions significantly improved the manuscript.

Sincerely

On behalf of all the authors

Rahel Mezemir.

Response to comments

1. The authors should revise the figures and tables as per the Journal submission guideline, and try to produce figures with good resolution

**Response**: The authors would like to thank the editors for their constructive comments and suggestions. The suggestion has well accepted the modification of the table and figure made according to PLoS one journal requirements.

2. Concerning the reference list, the authors changed the reference style from Vancouver to PLoS style in a word document in the menu of the reference style list .The authors did not use retracted paper in the reference list.
